# Follicular lymphoma regulatory T-cell origin and function

**DOI:** 10.3389/fimmu.2024.1391404

**Published:** 2024-05-10

**Authors:** Stéphane Rodriguez, Mehdi Alizadeh, Claire Lamaison, Alexis Saintamand, Céline Monvoisin, Rachel Jean, Laurent Deleurme, Jose Ignacio Martin-Subero, Céline Pangault, Michel Cogné, Patricia Amé-Thomas, Karin Tarte

**Affiliations:** ^1^ Unité Mixte de Recherche (UMR)1236, Université Rennes, INSERM, Etablissement Français du Sang Bretagne, Equipe Labellisée Ligue Contre le Cancer, Rennes, France; ^2^ Service Recherche, Etablissement Français du Sang, Rennes, France; ^3^ Pôle Biologie, Centre Hospitalier Universitaire, Rennes, France; ^4^ Univ Rennes, CNRS, INSERM, BIOSIT (BIOlogie, Santé, Innovation Technologique de Rennes) – Unité Mixte de Service 34 80, Rennes, France; ^5^ Departamento de Anatomía Patológica, Farmacología y Microbiología, Universitat de Barcelona, Barcelona, Spain; ^6^ Suivi Immunologique des Thérapeutiques Innovantes (SITI) Laboratory, Centre Hospitalier Universitaire Rennes, Etablissement Français du Sang Bretagne, Rennes, France

**Keywords:** follicular regulatory T cells, follicular helper T cells, follicular lymphoma, regulatory T cells, TCR repertoire

## Abstract

**Introduction:**

Follicular Lymphoma (FL) results from the malignant transformation of germinal center (GC) B cells. FL B cells display recurrent and diverse genetic alterations, some of them favoring their direct interaction with their cell microenvironment, including follicular helper T cells (Tfh). Although FL-Tfh key role is well-documented, the impact of their regulatory counterpart, the follicular regulatory T cell (Tfr) compartment, is still sparse.

**Methods:**

The aim of this study was to characterize FL-Tfr phenotype by cytometry, gene expression profile, FL-Tfr origin by transcriptomic analysis, and functionality by in vitro assays.

**Results:**

CD4^+^CXCR5^+^CD25^hi^ICOS^+^ FL-Tfr displayed a regulatory program that is close to classical regulatory T cell (Treg) program, at the transcriptomic and methylome levels. Accordingly, Tfr imprinting stigmata were found on FL-Tfh and FL-B cells, compared to their physiological counterparts. In addition, FL-Tfr co-culture with autologous FL-Tfh or cytotoxic FL-CD8^+^ T cells inhibited their proliferation *in vitro*. Finally, although FL-Tfr shared many characteristics with Treg, TCR sequencing analyses demonstrated that part of them derived from precursors shared with FL-Tfh.

**Discussion:**

Altogether, these findings uncover the role and origin of a Tfr subset in FL niche and may be useful for lymphomagenesis knowledge and therapeutic management.

## Introduction

Follicular lymphoma (FL) is the most common indolent B-cell lymphoma and corresponds to the immortalization of germinal center (GC) centrocytes (1, 2). Like their physiological counterparts, FL-B cells are dependent on a specific GC-like supportive microenvironment, illustrated by the selection of subclones harboring genetic alterations supporting their interactions with cells of the malignant niche (3, 4). As corollary, the FL microenvironment gene signature was demonstrated as more predictive of patient survival than FL-B-cell genetic alterations per se (5). Several mutations impact the crosstalk between FL-B cells and FL follicular helper T cells (Tfh) (6), including loss-of-function mutations of HVEM associated with an accumulation of Tfh overexpressing tumor necrosis factor-a (TNF) and lymphotoxin-a1b2 (LT), resulting in stromal cell activation (7).

Tfh are memory CD4^+^ T cells specifically expressing the transcription factor Bcl6. Upon differentiation, Tfh upregulate CXCR5, ICOS, and PD-1, which together modulate Tfh capacity to enter within the GC in response to CXCL13, where they support centrocyte selection and differentiation into plasma cells (8). FL-Tfh are prone to produce high amounts of IL-4 and IL-21 (9) involved in the polarization of tumor-associated macrophages (4), the recruitment of regulatory T cells (Treg) (10), and the survival and activation of FL-B cells in association with CD40L (11–13). In turn, FL-B/FL-Tfh crosstalk is believed to contribute to FL-Tfh expansion. In particular, gain-of-function mutations of the cysteine protease cathepsin S (CTSS) favor MHC II-restricted antigen presentation (3, 14) and increased Tfh infiltration in both mouse models and FL patients. In addition, clonal expansion is involved in Tfh expansion in FL (15).

Another key CD4^+^ T-cell subset commonly found at the periphery of malignant follicles is Treg. FL-Treg accumulate through the gradient of CCL19 and CCL22 chemokines produced by FL-B cells (10) and/or result from helper T cell (Th) differentiation toward the Treg lineage (16). The impact of Treg on FL disease and patient outcome was subjected to controversies (17, 18). Nevertheless, the prognostic value of FL Treg is believed to be related to their capacity to inhibit tumor-infiltrating cytotoxic cells (19). Actually, Treg populating FL malignant tissues are heterogeneous and can be split according to specific marker expression like GITR (20) or their spatial localization. Indeed, the intrafollicular pattern of FOXP3^pos^ cells was related to a poor prognostic, compared with the Treg diffuse pattern (21). This suggests that studying follicular Treg, rather than interfollicular Treg or whole Treg number, is more relevant in FL to decipher tumor pathogenesis.

Intrafollicular FOXP3^pos^ T cells were described as a specific Treg subset named follicular regulatory T cells (Tfr), sharing the expression of Treg (*Foxp3*, *CTLA-4*, *TNFRSF18*) and Tfh (*CXCR5*, *Bcl6*, *Icos*, *Pdcd1*) genes (22). Although Tfr were proposed to express IL1R2 and lack CD25 (*IL2Ra*) in humans (23), recent studies demonstrated that, depending on the Tfr differentiation stage, CD25 expression may vary (24, 25) and can be used to sort Tfr cells (26). In line with this, CD25^+^CXCR5^+^Foxp3^+^ CD4^+^ T cells assimilated to Tfr can be found in human peripheral blood (27) and lymph nodes (LNs) (28). Tfr were first thought to be exclusively derived from thymic Treg (22), with a self-specific repertoire able to control autoreactivity (29). However, additional studies highlighted an alternative origin by demonstrating Tfr differentiation from Foxp3^−^ CD4^+^ T cells (30). Additionally, Tfh may upregulate Foxp3 prior to GC contraction and showed an overlap of TCR repertoires between Foxp3^+^ and Foxp3^−^ GC T cells (31), and recently, a Tfh-to-Tfr developmental trajectory was identified in human tonsils (26). Tfr are required for optimal GC reaction as demonstrated in mice infection models (32, 33) and for immune response ending through their capacity to suppress both Tfh and GC B-cell activities (22, 25), partly through epigenetic changes (34). In FL, both Tfh and CXCR5^+^CD25^+^FOXP3^+^ Tfr are expanded, and their proportion among CD4^+^ T cells is correlated (13). Therefore, understanding the origin and role of FL-Tfr may be crucial to better understand lymphomagenesis.

In the current study, we revealed that FL-Tfr displayed a characteristic Treg phenotypic and transcriptomic profile with a highly demethylated *Foxp3*-CNS2 locus. TCR analysis indicated that a significant part of Tfr clones shares TCR Vb sequences with Tfh and, hence, a common progenitor. In addition, we showed that while FL-Tfr efficiently inhibit CD8^+^ T cells and Tfh *in vitro*, FL-Tfh support to FL-B cells was likely spared. Altogether, these results decipher the origin and critical roles of FL-Tfr within the niche and plead their targeting in the clinic to inhibit FL-B-cell progression.

## Materials and methods

### Cell samples

All tissues used for this study came from subjects recruited under institutional review board approval and informed consent process according to the Declaration of Helsinki. Human stromal cells from tonsils were obtained from pediatric patients undergoing routine tonsillectomy, as previously described (35). These cells were then cultured with 10% FCS in RPMI 1640 (Thermo Fisher, Waltham, USA) supplemented with penicillin and streptomycin. Lymphoid cells were recovered by perfusion of FL LN biopsies (*n* = 14) (**Supplementary Table 1**) or non-malignant tonsils (*n* = 12), enriched using Ficoll gradient and both magnetic and FACS sorting (see the procedure and gating strategies illustrated in the **Supplementary Material and Methods**).

### Flow cytometry characterization

**Figure 1:**
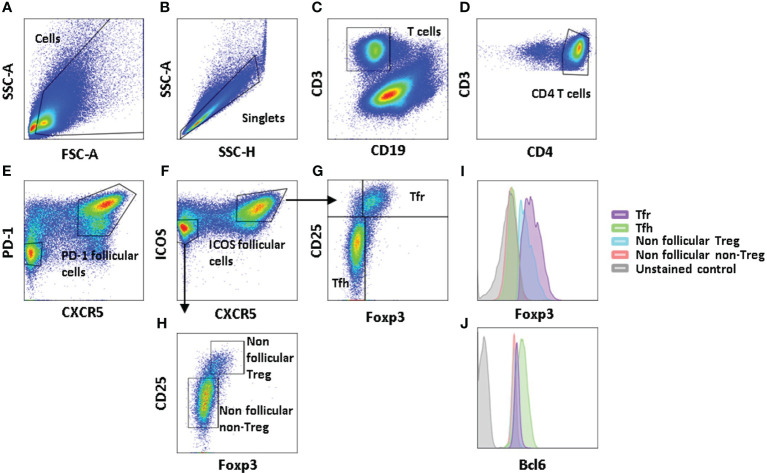
Gating strategy defining follicular CD4 and regulatory CD4 T cells. Illustrative example of the FL LN gating strategy: cells are gated according to FSC-A and SSC-A parameters **(A)**, and singlets are defined through SSC-H and SSC-A proportionality **(B)**. Dead cells and CD19-expressing cells are targeted within the same wavelengths and are removed together **(C)**. CD4 T cells are gated according to concomitant CD3 and CD4 expression **(D)**. Follicular CD4 T cells are defined through their dual CXCR5/PD-1 **(E)** or CXCR5/ICOS **(F)** expression, while non-follicular T cells are targeted as CXCR5^−^PD-1^−^ or CXCR5^−^ICOS^−^CD4 T cells. Regulatory T cells are gated as Foxp3^+^CD25^hi^CD4 T cells among follicular **(G)** or non-follicular **(H)** CD4 T cells. Illustrative histograms of Foxp3 **(I)** and Bcl6 **(J)** expression levels in Tfh, Tfr defined as follicular regulatory CD4 T cells, non-follicular Treg, non-follicular non-Treg CD4 T cells, and unstained control cells.

Samples were acquired on a Cytoflex^®^ flow cytometer (Beckman Coulter, Miami, FL, USA). Cell viability was determined by staining with live/dead fixable yellow dead cell stain kit (Thermo Fisher) prior to membrane marker staining and when required, fixation and permeabilization with eBiosciences Foxp3/Transcription Factor Staining Buffer Set (Life Technologies, Courtaboeuf, France). Cells were stained with antibodies referenced in **Supplementary Table 2**.** **A minimum of 100,000 cells per sample were acquired, and the gating strategy is illustrated in **Figure 1**. Subset analyses were done with FlowJo 10.4, LLC (Becton Dickinson, Franklin Lakes, USA), and the compensation matrix is provided in **Supplementary Table 3**. Statistical analysis was done with GraphPad Prism 6 software suite (GraphPad Software, La Jolla, CA, USA), the Mann–Whitney test was used, and standard deviations were illustrated.

### Microarray hybridization and data analyses

Microarray analyses were done on three samples of each following population: Tfh, non-follicular Treg, memory T, and naive T cells from the tonsils, as well as purified Tfh, Tfr, memory T, and naive T cells from FL LN, according to the gating strategies detailed in the **Supplementary Material and Methods**. RNA preparation and data preprocessing are also detailed in the **Supplementary Material and Methods**. Gene signatures were obtained using a *t*-test by comparison of memory T cells and each population of interest: Tfh or Tfr with limma package on R. To evaluate Tfr gene expression proximity to Tfh or Treg and activated Tfh to either FL-Tfh or tonsil Tfh, a pre-ranked Gene Set Enrichment Analysis [GSEA software and Molecular Signature Database (MSigDB)] was done. Classical GSEA was performed to determine the differentially enriched pathways between FL-Tfh and tonsil Tfh using the MSigDB hallmark collection and the resulting output to generate heatmaps (Heatmap.2 package). A similar GSEA strategy was used for centrocytes and FL-B-cell transcriptomic data previously published (36).

### Methylome analysis

TSDR analysis was performed with 500 ng of genomic DNA from FL LN naive Treg (nTreg), Tfr, and Tfh (*n* = 3) (the sorting strategies as technical and biological bias exclusion are detailed in the **Supplementary Material and Methods**), as previously described (37). The EZ DNA Methylation Kit (Zymo Research, Freiburg, Germany) was used for bisulfite conversion, and the bisulfite-converted DNA was hybridized onto the HumanMethylation 450K BeadChip kit (Illumina, Paris, France). Data from the 450k Human Methylation Array were analyzed in R using the minfi package (version: 1.6.0).

### Repertoire analysis

RNA was extracted from 150,000 cells of Tfh, Tfr, and Treg from FL LN using the Qiagen RNeasy Mini kit (Qiagen, Hilden, Germany). TCR sequences were amplified by unbiased RACE-PCR using the Clontech SMARTer RACE 5′/3′ kit (Clontech Laboratories, Inc., Mountain View, USA). Library preparation and the associated primer sequences are detailed in the **Supplementary Material and Methods**. Sequencing was done with a MiSeq Illumina and MiSeq Reagent Nano Kit v2. Pear software was used to match pair ends. FASTA sequences were then submitted to IMGT/HighV-Quest (http://www.imgt.org/) to align sequences with the reference database. Finally, clonotypes identified by IMGT were used to perform analysis with R packages (detailed analysis in the **Supplementary Material and Methods**). Circular graphics were done through the Circos package run on PERL.

### Proliferation assay

Tfh *in-vitro* survival was compromised without feeder cells, leading us to co-cultivate Tfh with tonsil stromal cells. In a round-bottom 96-well plate, 5,000 stromal cells were plated 1 day prior to Tfh seeding. Sorted Tfh were stained with CellTrace Far Red Kit (Thermo Fisher) to monitor their proliferation in culture. Either 60,000 Tfh or 40,000 Tfh + 20,000 Tfr were then plated in RPMI 1640 with 8% FCS (Thermo Fisher, Waltham, USA) and stimulated for 4 days with anti-CD3 (0.6 μg/ml) and anti-CD28 (0.2 μg/ml) stimulating antibodies (Sanquin, Amsterdam, Netherlands). On day 4, the supernatants were collected for cytokine quantification, while cells were trypsinized and stained with Annexin-V FITC, DAPI, and anti-CD7 BV711 allowing viable T-cell gating. Except for feeder cell usage, the same was done using CD8^+^ T cells stained with CellTrace Far Red. The proliferation index was calculated on the Annexin-V^−^ DAPI^−^ Cell Trace^+^ CD7^+^ population by ModFit LT software (Verity Software House, Topsham, USA).

### Cytokine quantification

To determine cytokine content in the co-culture supernatant, we used Luminex technology (Thermo Fisher, Waltham, MA, USA) with the MILLIPLEX kit: 7-Plex Human cytokine Mag kit comprising IFN-g, IL-4, IL-6, IL-10, sCD40L, MDC, and IL-21 (HSTCMAG-28SK) (Millipore, Fontenay Sous Bois, France), according to the manufacturer’s protocol. All cytokine concentrations were quantified within the same plate to avoid interexperimental variability. Results were processed with GraphPad. The Mann–Whitney test was used to determine significance.

### Immunofluorescence assay

Human tonsils, reactive LN, and FL LN were used (*n* = 3 for each tissue). FL grade 1–2 was selected, according to the World Health Organization criteria. Four-micrometer-thick whole-slide sections, obtained from FFPE tissue blocks, were processed by multiplex immunofluorescence staining with a U DISCOVERY 5-Plex IF. The staining and mounting procedures are detailed in the **Supplementary Material and Methods**. Visualization was performed with the NanoZoomer (Hamamatsu, Massy, France). FOXP3^+^/CD8^+^ and CD25^+^/CD8^+^ cell ratios were obtained in 10 follicles of each sample.

### Statistical analysis

All statistics were obtained from the GraphPad Prism 6 software suite (GraphPad Software, La Jolla, CA, USA). The statistical significance of the cytometry experiments was assessed through the non-parametric Wilcoxon test for matched pairs, and the Mann–Whitney test was used for unpaired data. *T*-test was used when a parametric test was required, and only two groups were tested for differential gene expression and to perform a correlation curve. When more than two groups had to be compared within cytometry or transcriptomic experiments, one-way ANOVA was done with Dunn’s multiple test correction or two-way ANOVA with Bonferroni post-test. Error bars correspond to the standard deviation. *, **, and *** correspond to *p* < 0.05, *p* < 0.01, and *p* < 0.005, respectively.

### Data sharing

Data are available on the GEO repository, with accession number GSE222532.

## Results

### Lymph node*-*related T*-*cell subset identification

To characterize CD4 T-cell subsets in FL, we first defined these different subpopulations in the tonsils and FL lymph nodes. Among CD4 T cells, we distinguished follicular cells expressing CXCR5 from non-follicular cells which are lacking it, with their discrimination being strengthened with either PD-1 (**Figure 1E**) or ICOS (**Figure 1F**) expression as an additional segregating marker. Regulatory T cells among follicular (**Figure 1G**) and non-follicular T cells (**Figure 1H**) were distinguished by their expression of Foxp3. Therefore, follicular T cells contain Tfh defined as CD3^+^CD4^+^CXCR5^hi^ICOS^+^Foxp3^−^ cells, which can be discriminated from their regulatory counterpart expressing Foxp3 (CD3^+^CD4^+^CD45RA^−^XCR5^+^ICOS^+^Foxp3^+^), while non-follicular T cells comprise classical Treg cells (CD3^+^CD4^+^CXCR5^−^ICOS^−^Foxp3^+^) and other memory T cells (CD3^+^CD4^+^CD45RA^−^CXCR5^−^Foxp3^−^).

### Expanded follicular Foxp3^+^ CD25^+^ subset in FL belongs to the Tfr population

**Figure 2:**
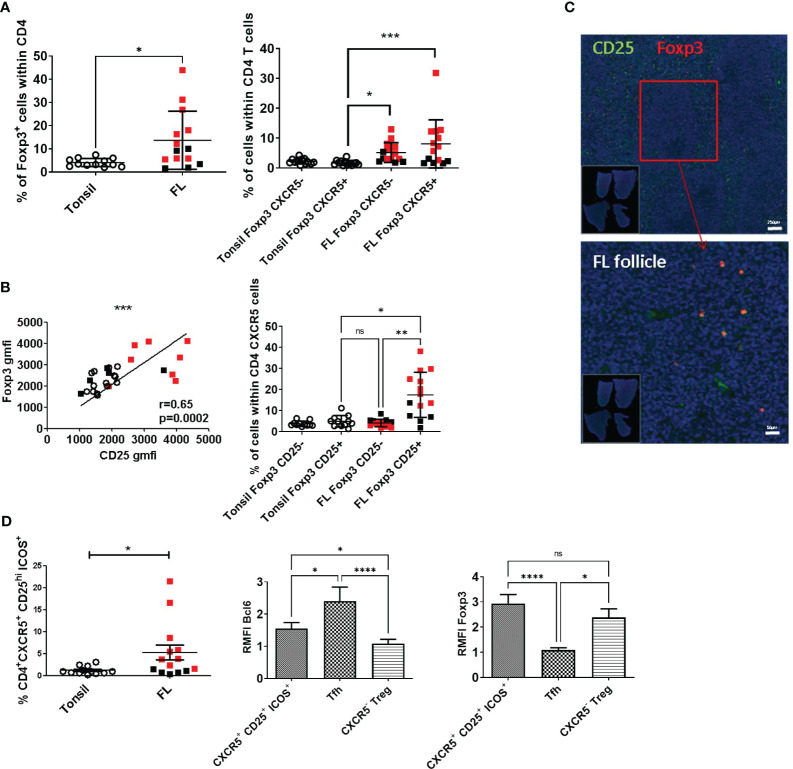
Phenotypical characterization of follicular regulatory T cells and *in-situ* localization. All over the figure, FL used in subsequent sorting experiments is depicted in red, and FL used only in cytometry experiments is depicted in black. **(A)** *Left*: The percentage of CD4^+^Foxp3^+^ cell number on CD4^+^ T cells was significantly higher (*p* = 0.0154) in FL (black and red squares, *n* = 14) than in tonsils (open circles, *n* = 12). *Right*: The frequency of CD4^+^Foxp3^+^CXCR5^+^ cells among the CD4^+^Foxp3^+^ population was significantly higher (*p* < 0.001) in FL than in tonsils. **(B)** *Left*: The correlation between Foxp3 and CD25 expression in CD4^+^ T cells was highly significant (*p* = 0.0002). *Right*: The percentage of CD25^+^ T cells within the CD4^+^Foxp3^+^CXCR5^+^ subset was significantly increased compared with the tonsil counterpart. **(C)** *Upper panel*: CD25 (green) Foxp3 (red) staining on FFPE FL samples. *Lower panel*: Higher magnification of the FL sample focused on the follicular area highlighted cells co-expressing CD25 and Foxp3 within the malignant follicle. **(D)** *First panel*: The frequency of CD4^+^CXCR5^+^CD25^hi^ICOS^+^ T cells was significantly increased in FL compared with tonsils (*p* = 0.0276). The ratio of mean of fluorescence intensity of Bcl6 (*middle panel*) and Foxp3 (*left panel*) expression normalized on non-follicular non-Treg T-cell marker expression. Tfr and Tfh cells expressed significantly more Bcl6 than non-follicular Treg (Tfr vs. Treg: *p* < 0.05; Tfh vs. Treg: *p* < 0.0001). Tfr and Treg expressed a significantly higher level of Foxp3 than Tfh (Tfr vs. Tfh: *p* < 0.001; Treg vs. Tfh: *p* < 0.05). *p<0.05, **p<0.01, ***p<0.005, ****p<0.0001, ns, not significant.

Since different T-cell subsets were found participating in FL maintenance and the role of regulatory T cells in this context is still ambiguous, we carefully analyzed CD4^+^ regulatory T-cell subsets from malignant LN (*n* = 14) and compared them with their tonsil counterpart (*n* = 12). We found a significant expansion of the Foxp3^+^ subset among CD4^+^ T cells in FL LN (*p* = 0.015, mean tonsil = 4.3 ± 0.499%, mean FL black and red squares = 13.7 ± 3.3%) (**Figure 2A**, left). This expansion relied on the significant amplification (*p* < 0.001) of cells harboring a Tfr phenotype, i.e., Foxp3^+^CXCR5^+^CD4^+^ T cells, rather than Foxp3^+^ CXCR5^−^ corresponding to non-follicular Treg (mean tonsil CD4^+^Foxp3^+^CXCR5^+^ = 1.91 ± 0.206%, mean tonsil CD4^+^Foxp3^+^CXCR5^+^ = 1.695 ± 0.834%, mean FL CD4^+^Foxp3^+^CXCR5^−^ = 5.16 ± 3.313%, mean FL CD4^+^Foxp3^+^CXCR5^+^ = 8.07 ± 8.057%; **Figure 2A**, right). A strong correlation between Foxp3 and CD25 expression was observed in both tonsil and FL LN CD4^+^ T cells (*p* < 0.001, **Figure 2B**, left), and the CD25^+^ subpopulation was the major contributor to the CXCR5^+^Foxp3^+^ FL CD4^+^ T-cell expansion (mean tonsil CD4^+^Foxp3^+^CXCR5^+^CD25^+^ = 4.944 ± 2.738%, mean FL CD4^+^Foxp3^+^CXCR5^+^CD25^+^ = 17.50 ± 10.69%, mean FL CD4^+^Foxp3^+^CXCR5^+^CD25^−^ = 4.134 ± 1.765%; **Figure 2B**, right). In agreement, we validated by immunofluorescence the presence of follicular Foxp3^+^CD25^+^ cells within FL malignant follicles (**Figure 2C**). The addition of ICOS to the CD4, CXCR5, and CD25 markers demonstrated a better efficiency than PD1 to retrieve follicular CD4^+^ T cells expressing Foxp3 in FL (**Supplementary Figure 1A**). Accordingly, this CD4^+^CXCR5^+^CD25^+^ICOS^+^ population was found expanded in FL (**Figure 2D**, left; mean tonsils = 1.188 ± 0.905%, mean FL = 5.377 ± 6.61%, *p* = 0.028). To assert the Tfr phenotypical identity of this subset, we explored its Bcl6 expression and found that it was significantly higher in Tfr (RMFI: Tfr/CXCR5^+^CD25^+^ICOS^+^ T cells = 1.5 ± 0.32, Tfh cells = 2.2 ± 0.46) than in CXCR5^−^ non-follicular Treg (non-follicular Treg = 1 ± 0.059) (**Figure 1D**, middle; *p* < 0.05), while Foxp3 expression was similar in the two regulatory subsets (**Figure 2D**, right; RMFI: Tfr/CXCR5^+^CD25^+^ICOS^+^ T cells =2.937 ± 0.3547, non-follicular Treg = 2.383 ± 0.3376) compared with the decreased one of Tfh (Tfh = 1.087 ± 0.092). Altogether, these indicated that FL-Tfr can be sorted for transcriptomic and functional studies based on their CD25 and ICOS high expression.

### FL-Tfr display a regulatory gene profile

**Figure 3:**
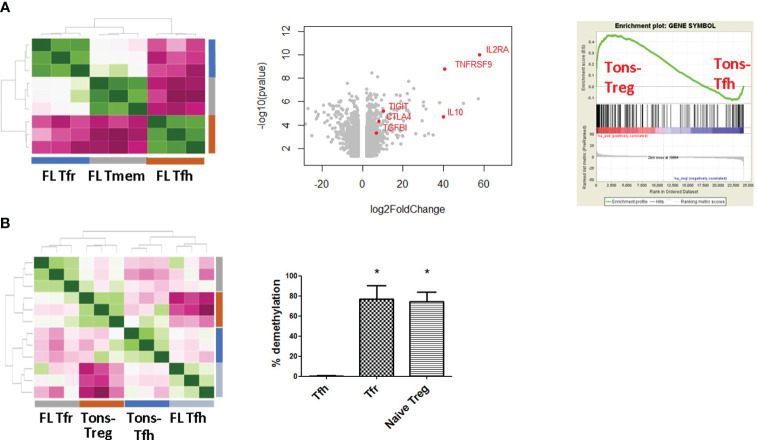
FL-Tfr are close to Treg at the gene level. FL-Tfh, FL memory T cells, FL-Tfr, and tonsil non-follicular CXCR5^−^ Treg were sorted (*n* = 3 for each population) based on CD4, CXCR5, CD25^hi^, and ICOS marker expression. **(A)** *Left panel*: Correlation heatmap of Tfh, Tfr, and memory T cells. All samples clusterized according to their lineage. Tfh and Tfr clustered among distinct groups. *Middle panel*: Volcano plot of differentially expressed genes (log FC ≥2, *p* < 0.05) between memory T cells (genes on the left) and Tfr (genes on the right). Genes related to regulatory function were depicted in red. *Right panel*: FL-Tfr transcriptomic profile compared with tonsil Tfh and non-follicular Treg by GSEA. Tfr are significantly enriched (*p* < 0.0001) with Treg genes. **(B)** *Left panel*: Bisulfite sequencing methylome correlation heatmap of tonsil Tfh and non-follicular Treg and FL-Tfh and Tfr. Tfh samples cluster together independently of their FL or tonsil origin, while Treg and FL-Tfr cluster together. *Right panel*: TSDR (Foxp3 CNS2 locus) DNA in FL-Tfh, FL-Tfr, and FL naive Treg (*n* = 5) was found significantly demethylated (*p* < 0.05) in both and in naive Treg compared with Tfh. *p<0.05.

To get deeper insights in the FL-Tfr profile, CD4^+^CXCR5^+^CD25^+^ICOS^+^ FL-Tfr (*n* = 3) were sorted from selected FL LN samples where more than 90% of these cells expressed Foxp3 (depicted in red in **Figure 2**, description of the corresponding patients in **Supplementary Table 1**) and their transcriptomic profiles were compared with those of FL-Tfh (*n* = 3), FL-CXCR5^−^Foxp3^−^ memory CD4^+^ T cells (FL-Tmem) (*n* = 3), inflamed tonsil (Tons)-derived Tfh (Tons-Tfh) (*n* = 3), Tons-non-follicular Treg (Tons-Treg) (*n* = 3), and Tons-Tmem (*n* = 3). FL T cells clustered according to their subset of origin (**Figure 3A**, left, and **Supplementary Figure 2A**). Tonsil-non-follicular Tmem was used as a reference population to highlight either Tfh- or Tfr-specific features as characteristics related to their physiological or malignant microenvironment. Comparison of FL-Tfr with cells highlighted the regulatory phenotype of FL-Tfr with high levels of *IL2RA*, *IL10*, *CTLA-4*, *Foxp3*, and *TIGIT* transcripts (**Figure 3A**, middle). Furthermore, GSEA revealed that the FL-Tfr transcriptomic profile was enriched for genes expressed by Tons-Treg, like *IL2RA*, *CTLA-4*, and *Foxp3*, rather than genes expressed by Tons-Tfh (*p* < 0.0001) (**Figure 3A**, right). In addition, we found a significant overlap (*p* = 1.025 e^−21^) between the FL-Tfr transcriptomic signature and the murine Tfr signature (22) (**Supplementary Figure 2B**). Finally, FL-Tfr clustered with Tons-Treg in the methylome analysis (**Figure 3B**, left), in agreement with their preferential demethylation of *Foxp3*, *Ctla-4*, *Tnfrsf9*, and *Ikzf4*, in comparison with FL-Tmem or FL-Tfh (**Supplementary Figure 2C**). Finally, we studied *Foxp3* CNS2 demethylation status, a marker of all Treg subsets regardless of their thymic or peripheral origin (37), in FL-Tfr (**Figure 3B**, right) (*n* = 3). As expected, *Foxp3* CNS2 of FL-Tfh was found poorly demethylated (mean = 0.58 ± 0.51%), while demethylation was high and similar in FL-Tfr (mean = 75.6 ± 9.94%), and FL I CD45RA^+^ Treg was used as a control for thymic Treg (mean = 74.08 ± 8.51%), supporting the regulatory lineage affiliation of FL-Tfr. Altogether, these data demonstrate that in FL, follicular CD4^+^ T cells co-expressing CD25 and ICOS correspond to Foxp3^+^Bcl6^+^ Tfr cells. At both the transcriptomic and methylome levels, these FL-Tfr display a regulatory phenotype similar to Treg.

### Tfr share the TCR repertoire with both Tfh and CXCR5*^−^* Treg in FL samples

**Figure 4:**
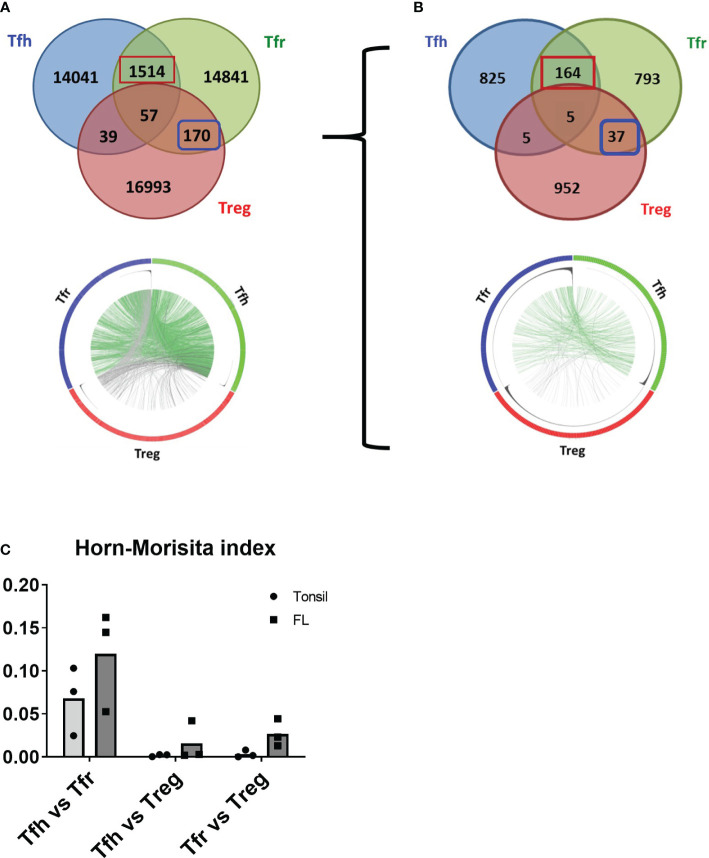
TCRvβ repertoire analysis of FL T cells. **(A, B)** Venn diagrams demonstrating the overlap between Tfh (blue circle), Tfr (green circle), and Treg (red circle) from a representative sample (*n* = 3). Numbers within circles represent the number of clones identified per population. Numbers at the circle intersections represent clones shared between the related populations. The *left Venn diagrams* illustrate all clones identified, while the *right Venn diagrams* represent the 1,000 most abundant clones for each population. Circles in the lower part of the figure (Circos) represent subset repertoire size, clone frequencies, and community over the studied populations, each Circos being related to its upper Venn diagram. The outer circle contains subset-related clones colored according to Venn diagram colors. The middle circle represents clone frequency histograms. The inner circle figures the clone community between T-cell populations with lines in green when the clones are shared between Tfh and Tfr and lines in gray for other communities. **(C)** The Horn–Morisita index illustrates the repertoire overlap for each comparison.

To determine whether FL-Tfr originate from thymic Treg and/or from the peripheral differentiation of activated CD4^+^ T cells, we performed TCR repertoire analysis of different CD4^+^ T-cell subsets populating FL malignant LN (*n* = 3). FL-Tfh, FL-Tfr, and FL-non-follicular Treg were sorted, and their TCR-Vβ were sequenced following an unbiased TCR-Vβ sequence amplification by RACE-PCR. Approximately 150,000 cells per subset were sorted from each FL sample, allowing to collect 14,000 to 28,000 clones with a homogeneous repartition among subsets and clone frequencies ranging from 1 to 2,000. Although a low number of clones were shared between Tfh and Treg or even by all cell subsets, a more robust repertoire community was observed between both Tfr and Tfh, as well as between Tfr and Treg (**Figure 4A**). Remarkably, clones shared between FL-Tfh and FL-Tfr were found among the most frequent clones (**Figure 4A**, Circos plot, clones frequency is represented as black histograms), suggesting that they resulted from antigen-driven amplification of a common precursor or their polarization toward an alternative phenotype (Tfh or Tfr). In line with this result, an overlap between FL-Tfr and FL-Tfh (FL-Tfh/FL-Tfr overlap = 5%) was found almost doubling when the analysis was restricted to the 1,000 most frequent clones (FL-Tfh/FL-Tfr overlap = 9%) (**Figure 4B**, up and down). Furthermore, for both the FL and tonsil samples, the Horn–Morisita index, which illustrates the repertoire degree of similarity, was found higher for Tfh/Tfr than for Tfr/Treg, while the Tfh/Treg overlap was null as expected (**Figure 4C**). Altogether, these results identified Tfr as clonally related primarily to Tfh but also to Treg, particularly in FL. Whether this differential origin has an impact on their functional activity still remains to be explored.

### Tfr are functional in the FL microenvironment

**Figure 5:**
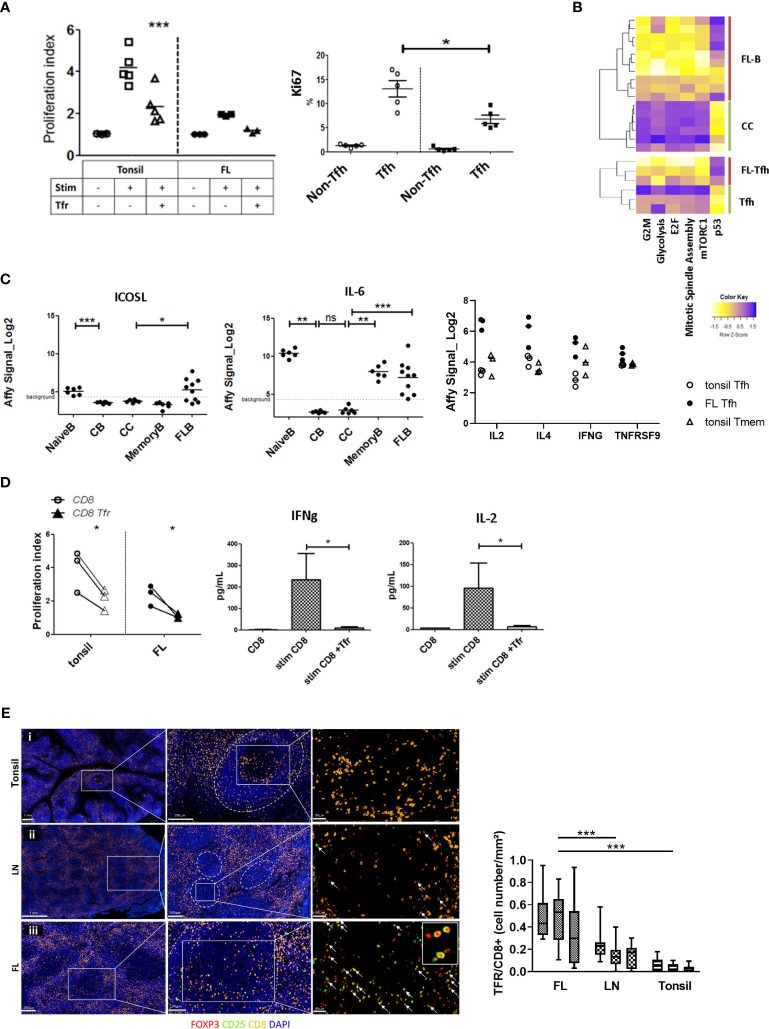
Assessment of FL-Tfr impact on FL-Tfh and malignant B cells. **(A)** *Upper panel*: Tfh and Tfr from the tonsils (*n* = 5) (open symbols) or FL (*n* = 3) (filled symbols) were sorted for the co-culture experiments. The proliferation index of resting Tfh (circles: Tfh), anti-CD3/CD28 activated Tfh (squares: Act Tfh), or anti-CD3/CD28 activated Tfh co-cultured with Tfr (triangles: Act Tfh +Tfr) is shown. Tfr addition significantly inhibited (*p* < 0.001) tonsil Tfh proliferation but not the proliferation of FL-Tfh. *Lower panel*: The percentage of Ki67^+^ cells in tonsil non-Tfh, tonsil Tfh (open circles), and FL non-Tfh, FL-Tfh (filled squares) among T cells. The percentage of Ki67^+^ cells was significantly lower in FL-Tfh compared with tonsil Tfh (*p* < 0.05). **(B)** Heatmaps illustrating enriched GSEA pathways in FL or tonsil Tfh and tonsil centrocytes (CC) or FL-B cells. **(C)** *ICOSL* (*left panel*) and *IL6* (*middle panel*) transcriptomic probe set intensities in tonsil naive B, tonsil centroblast (CB), tonsil centrocyte (CC), tonsil memory B, and FL-B-cell subsets. *ICOSL* and *IL6* are significantly upregulated in FL-B cells compared with CC (*p < 0.05, **p < 0.01, ***p < 0.001). *Right panel*: T-cell subset expression levels of characteristic genes of interest, significantly upregulated in FL-Tfh (closed symbols) compared with tonsil CD4 subsets (open symbols). **(D)** *Left panel*: Tonsil CD8^+^ T cells (empty symbols) or FL CD8^+^ T cells (filled symbols) stimulated with anti-CD3/CD28 were cultured with (triangles) or without (circles) autologous Tfr. Tfr addition to CD8^+^ T cells from both tonsils or FL decreased significantly (*p* < 0.05) CD8^+^ T-cell proliferation (*n* = 3). Culture supernatant quantification of IFN-γ (*middle panel*) and IL-2 (*right panel*). Tfr addition to stimulated CD8^+^ T-cell culture led to a significant (*p* < 0.05) decrease of IL-2 concentrations, compared with CD8 cultured without Tfr (*n* = 3). **(E)** *Left panel*: CD8^+^ and CD25^+^FOXP3^+^ spatial cell organization in the tonsil (*i*), lymph node (*ii*), and follicular lymphoma lymph node follicles (*iii*). GCs were outlined with white dotted lines. Tfr were assumed to be characterized by concomitant CD25 (green) and FoxP3 (red) staining associated with follicular localization. CD8^+^ cells were stained yellow and nucleus (DAPI) blue. In FL, more Tfr were observed, while CD8^+^ cells were not increased. *Right panel*: Boxplot of Tfr/CD8^+^ T-cell ratio in FL, reactive lymph node (LN), and tonsils (*n* = 3 for each) by immunofluorescence count of 10 follicles per sample. Tfr/CD8^+^ T-cell ratio was significantly increased in FL compared with reactive lymph node. ****p<0.0001, ns, not significant.

Physiologically, Tfr are known to modulate Tfh and GC-B-cell responses. In the FL context, Tfr functions have not yet been estimated. Autologous culture of sorted Tfr and Tfh from the tonsils or FL LN was thus performed in the presence of anti-CD3/-CD28 stimulation (*n* = 3). As expected, the proliferation of activated tonsil Tfh was significantly reduced (*p* < 0.001) in the presence of Tons-Tfr. A similar trend was observed when FL-Tfh were co-cultured with FL-Tfr (**Figure 5A**, top). Interestingly, *in-vitro* stimulated FL-Tfh displayed a lower proliferation index than Tons-Tfh, and the addition of FL-Tfr abrogated the residual proliferative capacity of FL-Tfh. This finding was in line with the significantly lower percentage of KI67^+^ cells found within *ex-vivo* FL-Tfh (6.796 ± 1.963%) compared with Tons-Tfh (13.06 ± 0.33%) (*p* < 0.05) (**Figure 5A**, bottom), and the specific downregulation of pathways related to cell cycle and growth (**Figure 5B**, left part) was investigated by the transcriptomic approach. mTORC1 and glycolysis pathways were also found downregulated in FL-Tfh, matching the signature of *in-vitro* murine Tfh suppressed by Tfr (34). These data argue for a FL-Tfh regulation by FL-Tfr *in vivo*. Of note, the same pathways were found downregulated in FL-B cells compared with healthy centrocytes (**Figure 5B**, right part), hypothesizing that FL-B cells were subjected to the same regulatory environment as FL-Tfh. However, both FL-Tfh and FL-B cells exhibited an increased effector profile. The *ICOSL* and *IL-6* genes were found highly expressed in FL-B cells (**Figure 5C**, left and middle, respectively), while FL-Tfh exhibited more *IL-4*, *IL-2*, *IFN-g*, and *TNFRSF9* mRNAs than their respective tonsil counterparts (**Figure 5C**, right). Since the prognostic value of Treg in FL has been linked to their capacity to inhibit tumor-infiltrating cytotoxic CD8^+^ T cells (19), we decided to study the function of CD8^+^ T cells sorted from either the tonsils or FL LN. Anti-CD3/-CD28 antibody stimulation induced CD8^+^ T-cell proliferation (**Figure 5D**, left, circles) (*n* = 3). Nevertheless, FL CD8^+^ T cells exhibited a lower proliferation index than tonsil CD8^+^ T cells. The addition of autologous Tfr significantly reduced the proliferation capacity of CD8^+^ T cells in both tonsil and FL contexts (*p* < 0.05) (**Figure 5D**, left, triangles). In accordance, less IFN-γ (mean CD8 = 233 ± 122.3 pg/ml vs. mean CD8+Tfr = 8.857 ± 6.869 pg/ml) and IL-2 Tfr (mean CD8 = 95.56 ± 57.73 pg/ml vs. mean CD8 + Tfr = 6.527 ± 2.797 pg/ml) were found in the supernatant of activated CD8^+^ T cells co-cultured with FL-Tfr (**Figure 5D**, middle and right). Finally, to confirm that FL-Tfr indeed impacted CD8^+^ T cells *in vivo*, we assessed intrafollicular Foxp3^+^CD25^+^/CD8^+^ cell ratio in FL LN in comparison to reactive LN and inflamed tonsils (*n* = 3). We observed a significant increase (FL: 0.45 ± 0.089, reactive LN: 0.17 ± 0.05, tonsil: 0.04 ± 0.02, *p* < 0.0001) of the intrafollicular Foxp3^+^CD25^+^/CD8^+^ cell ratio in FL LN samples (**Figure 5E**), and a similar trend was observed by flow cytometry (**Supplementary Figure 3**), strengthening our hypothesis of a CD8^+^ T-cell control by FL-Tfr *in vivo*. Therefore, in light of these results, FL-Tfr likely exert their regulatory function on lymphoid cell proliferation in malignant follicles, but FL-Tfh and B cells somehow escape Tfr functional control in contrast to CD8^+^ T cells.

## Discussion

In this study, we deciphered CXCR5^+^ regulatory T-cell compartment in FL. We confirmed that CXCR5^+^Foxp3^+^CD4^+^ T cells were more frequent in FL and demonstrated that this population was specifically enriched within CD25^hi^ICOS^+^CXCR5^+^ cells. Although ICOS has already been described on Tfr (13), CD25 downregulation is required to allow its differentiation from extrafollicular CD25^+^ Treg, and this phenotype is classically used to distinguish Tfr from classical Treg (32, 38). Nevertheless, recent articles demonstrated in mice that i) Tfr populations differentially expressing CD25 co-exist in follicles, ii) CD25^+^ Tfr displaying Bcl6 expression are found at the GC periphery (25), and iii) the CD25–STAT5 axis is associated with Tfr lineage maintenance and optimal GC reaction regulation (39). Since we found the CD25^hi^CXCR5^+^ICOS^+^CD4^+^ T-cell subset while expressing a similar Foxp3 level than non-follicular Treg expressed more Bcl6 and that amplification of CD25^+^ Foxp3^+^ T cells occurs *in situ* within FL follicles, we assumed that FL CD25^hi^CXCR5^+^ICOS^+^CD4^+^ T cells belong to the Tfr lineage. Transcriptomic and methylome data confirmed the Tfr profile of these cells but did not question their origin in FL.

Although the Tfr origin was primarily attributed to thymic Treg (22, 29), several mouse studies demonstrated that Tfr can also emerge from peripheral differentiation of I CD4^+^ T cells (15, 30, 31). Given that we previously found a correlation between enrichments of paired FL-Tfr and Tfh (13), we hypothesized a peripheral origin for most FL-Tfr. To answer this question, a TCRvβ mRNA sequencing was performed, and the repertoires of FL-Tfh, total Treg, and Tfr were compared. Although a large fraction of FL-Tfr clones was not assigned to either Tfh or Treg repertoires, the highest overlap was observed between Tfr and Tfh repertoires. This result underlines that i) at least a part of FL-Tfr shares common precursors with Tfh or is derived from each other, ii) the origin of these clones cannot be from thymic Treg, and iii) the repertoire of these clones should be directed against foreign Ag. These observations together with the overlap between Tfr and Treg repertoires plead for a multiple origin of Tfr in FL and reconcile studies demonstrating that Tfr may root from non-follicular CD4^+^ precursors (30), directly from pre-Tfh and/or already differentiated GC-Tfh (31) and/or from thymic (22) or induced Treg (30). Actually, FL-B cells support Tfh subset expansion (3, 6), Treg expansion through Treg recruitment by the production of CCL17 and CCL22 (10), Th17 skewing toward Treg (16), or Treg induction through ICOSL (40). Therefore, FL-B cells may actively contribute to the diversity of the origin of Tfr through the amplification of precursor pools.

Furthermore, whether the origin of Tfr is related to its TCR-associated Ag and signal induction in the FL context needs to be elucidated. As the FL-Tfr origin is multiple, their activation and function may differ depending on this factor. To study Tfr activity *in vitro*, classical CD3/CD28 stimulation was used, and the impact of Tfr on autologous Tfh and CD8^+^ cell proliferation was demonstrated. Interestingly, studying FL-Tfh and FL-B-cell gene signatures revealed a putative phenotype of Tfr imprinting, as found in mouse Tfh and B cells inhibited by Tfr (34). Nevertheless, the decreased proliferation ability of FL-Tfh was associated with an already described activation signature (IFN-g, IL-4, and TNF-a upregulation (13)), concomitant with an increased expression of IL-6 and ICOS-L by FL-B cells. These findings contrast with Tfr-mediated CD8^+^ T-cell inhibition, which results in both decreased CD8^+^ T-cell proliferation and activity (decreased IFN-g production). Whether this incomplete FL-Tfh and FL-B-cell-inhibited profile is related to a defective interaction between Tfr and their targets or involves other mechanisms has to be determined. In FL, most patients display a malignant B-cell monoclonal profile with poorly proliferative B cells compared with centrocytes, and whether this cycling defect is solely related to Bcl2 overexpression (41) or to any FL-Tfr impact as we observed at the transcriptomic level has to be determined. In the context of functional FL-Tfr, the resistance of FL-B cells to Tfr regulation may be explained by one or several of the following reasons: i) B-cell clones frequently exhibit a decreased capacity to present antigen due to mutation of *CREBBP* (42), potentially impacting FL-B cells/FL-Tfr immune synapse formation. ii) The mTOR pathway of FL-B cells, which is disturbed by Tfr imprinting, is mutated in 10% to 15% of FL cases (43, 44). Iii) The FL microenvironment favors Tfh-mediated support to FL-B cells: FL-B-cell mutations of HVEM (7), their high CD40 (45) expression, the IL-21 (34)-rich environment related to both FL-Tfh amplification, and the differential FL-Tfr origin with Tfh-derived Tfr retention of IL-21 production capacity (26) may explain FL-Tfr differential impact on FL-B cells, FL-Tfh, and CD8^+^ T cells.

Altogether, these findings uncover the role of Tfr in the FL niche and may be useful to gain knowledge on lymphomagenesis and its therapeutic management. Recently, anti-PD-1 was tested in the clinic to treat relapsing FL patients (46) as well as patients bearing renal cell carcinoma, melanoma, or non-small lung carcinoma (47). This study interrogates about the impact of such treatment on the FL niche, since both Tfh and Tfr express high levels of PD-1, in addition to infiltrating CD8^+^ cytotoxic T cells. Further studies are required to decipher this observation.

## Data availability statement

The datasets presented in this study can be found in online repositories. The names of the repository/repositories and accession number(s) can be found below: https://www.ncbi.nlm.nih.gov/geo/, GSE222532.

## Ethics statement

The studies involving humans were approved by the Ethics Committee of Rennes Hospital. The studies were conducted in accordance with the local legislation and institutional requirements. The human samples used in this study were acquired from diagnostic biopsies. Written informed consent for participation was not required from the participants or the participants’ legal guardians/next of kin in accordance with the national legislation and institutional requirements.

## Author contributions

SR: Conceptualization, Data curation, Formal analysis, Funding acquisition, Investigation, Methodology, Software, Validation, Writing – original draft, Writing – review & editing. MA: Data curation, Formal analysis, Investigation, Methodology, Software, Validation, Writing – review & editing. CL: Data curation, Formal analysis, Investigation, Methodology, Software, Visualization, Writing – review & editing. AS: Data curation, Formal analysis, Investigation, Methodology, Software, Writing – review & editing. CM: Investigation, Methodology, Validation, Writing – review & editing. RJ: Investigation, Methodology, Validation, Writing – review & editing. LD: Investigation, Methodology, Writing – review & editing. JM-S: Conceptualization, Data curation, Formal analysis, Investigation, Methodology, Software, Validation, Writing – review & editing. CP: Data curation, Formal analysis, Investigation, Methodology, Software, Writing – review & editing. MC: Conceptualization, Methodology, Software, Supervision, Validation, Writing – review & editing. PA-T: Conceptualization, Data curation, Formal analysis, Funding acquisition, Investigation, Methodology, Project administration, Resources, Software, Supervision, Validation, Visualization, Writing – original draft, Writing – review & editing. KT: Conceptualization, Data curation, Formal analysis, Funding acquisition, Investigation, Methodology, Project administration, Resources, Software, Supervision, Validation, Visualization, Writing – original draft, Writing – review & editing.
