# Corrigendum: Follicular lymphoma regulatory T-cell origin and function

**DOI:** 10.3389/fimmu.2024.1472373

**Published:** 2024-08-15

**Authors:** Stéphane Rodriguez, Mehdi Alizadeh, Claire Lamaison, Alexis Saintamand, Céline Monvoisin, Rachel Jean, Laurent Deleurme, Jose Ignacio Martin-Subero, Céline Pangault, Michel Cogné, Patricia Amé-Thomas, Karin Tarte

**Affiliations:** ^1^ Unité Mixte de Recherche (UMR)1236, Université Rennes, INSERM, Etablissement Français du Sang Bretagne, Equipe Labellisée Ligue Contre le Cancer, Rennes, France; ^2^ Service Recherche, Etablissement Français du Sang, Rennes, France; ^3^ Pôle Biologie, Centre Hospitalier Universitaire, Rennes, France; ^4^ Univ Rennes, CNRS, INSERM, BIOSIT (BIOlogie, Santé, Innovation Technologique de Rennes) – Unité Mixte de Service 34 80, Rennes, France; ^5^ Departamento de Anatomía Patológica, Farmacología y Microbiología, Universitat de Barcelona, Barcelona, Spain; ^6^ Suivi Immunologique des Thérapeutiques Innovantes (SITI) Laboratory, Centre Hospitalier Universitaire Rennes, Etablissement Français du Sang Bretagne, Rennes, France

**Keywords:** follicular regulatory T cells, follicular helper T cells, follicular lymphoma, regulatory T cells, TCR repertoire

In the published article, there was an error. An acknowledgment has been forgotten.

A correction has been made to the **Acknowledgments** section. This section previously stated:

“The authors are thankful to the Centre de Ressources Biologiques (CRB)-Sant́e (BB-0033-00056, http://www.crbsante-rennes.com) of Rennes Hospital for its support in the processing of biological samples, the Clinique La Sagesse and Christophe Ruaux for providing the tonsil samples, and the UMS CNRS 3480/US INSERM 018 BIOSIT for the cell sorting core facility. The authors thank Violaine Alunni from the IGBMC Microarray and Sequencing platform (Illkrich Graffestaden, France) for Affymetrix GeneChip analysis. The authors thank Birgitt Sawitzki (Charity Hospital, Berlin, Germany) for TSDR methylation assessments.”

The corrected **Acknowledgments** section appears below:

“This work was realized in the context of the LabEX IGO program (n° ANR-11-LABX-0016) funded by the «Investment into the Future» French Government program, managed by the National Research Agency (ANR). The authors are thankful to the Centre de Ressources Biologiques (CRB)-Sant́e (BB-0033-00056, http://www.crbsante-rennes.com) of Rennes Hospital for its support in the processing of biological samples, the Clinique La Sagesse and Christophe Ruaux for providing the tonsil samples, and the UMS CNRS 3480/US INSERM 018 BIOSIT for the cell sorting core facility. The authors thank Violaine Alunni from the IGBMC Microarray and Sequencing platform (Illkrich Graffestaden, France) for Affymetrix GeneChip analysis. The authors thank Birgitt Sawitzki (Charity Hospital, Berlin, Germany) for TSDR methylation assessments.”

The authors apologize for this error and state that this does not change the scientific conclusions of the article in any way. The original article has been updated.

