# Supplementary figures and images for: Follicular lymphoma regulatory T-cell origin and function
Source: Front Immunol. 2024 May 10;15:1391404. doi: 10.3389/fimmu.2024.1391404 (PMC11116630; doi:10.3389/fimmu.2024.1391404)

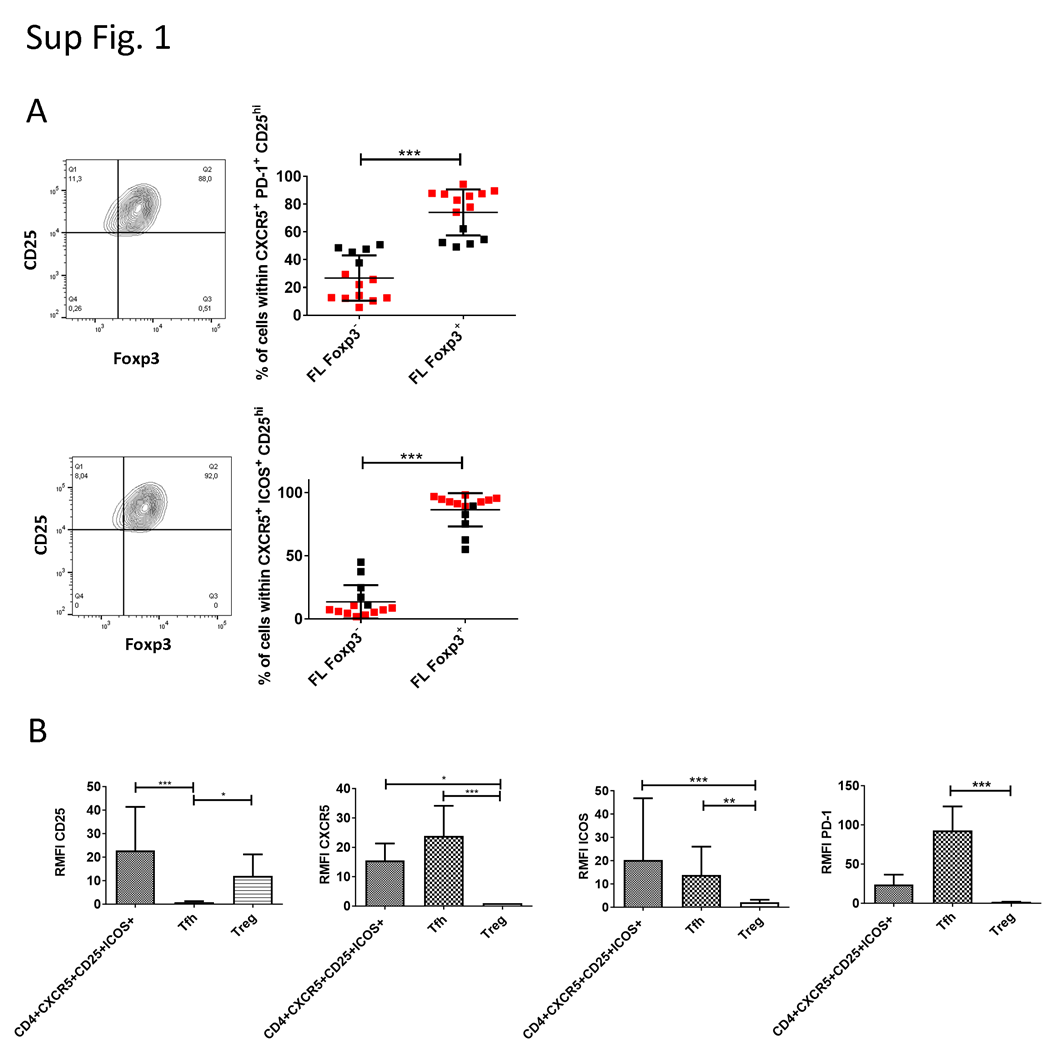

Supplement: Supplementary file 1 [file Image_1.png]

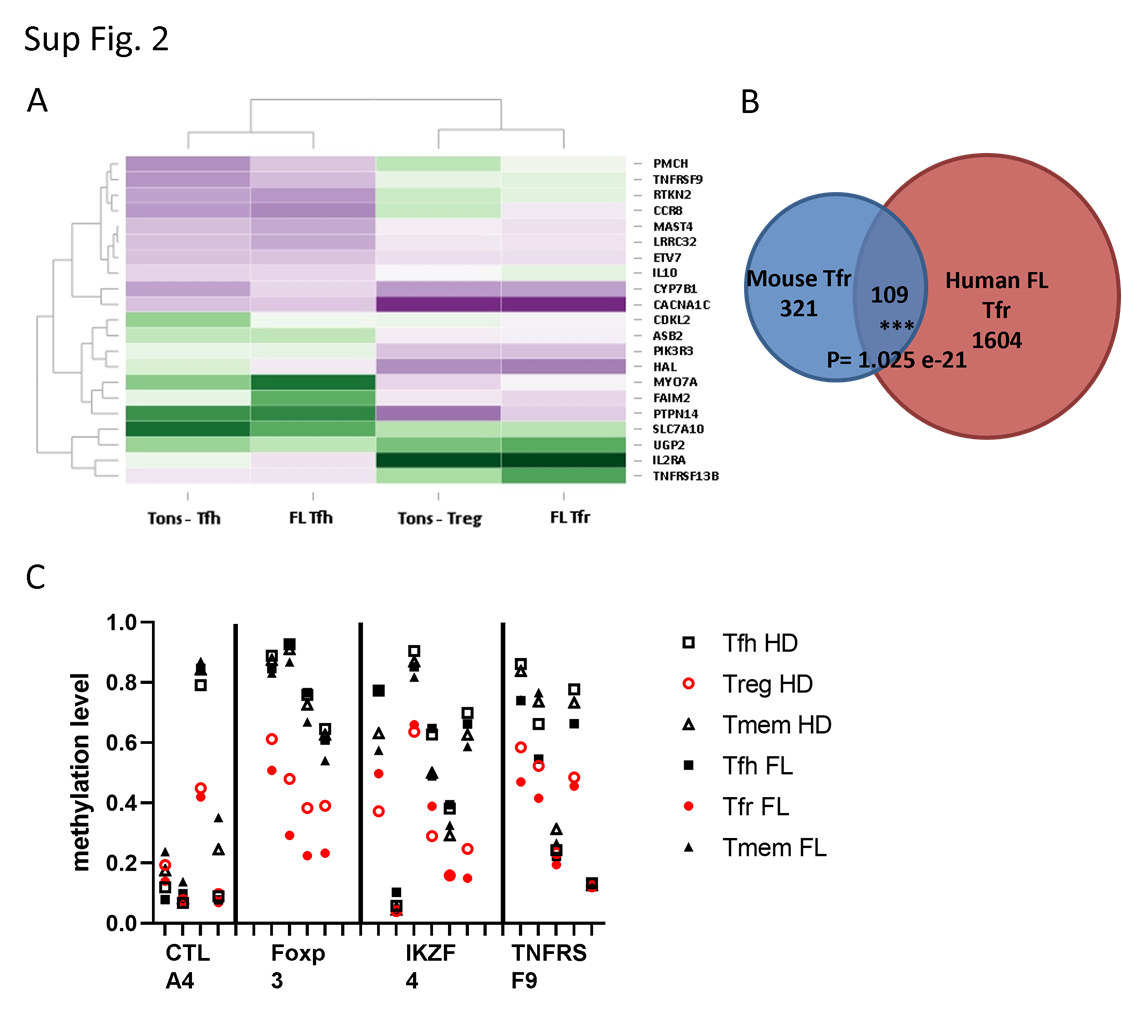

Supplement: Supplementary file 2 [file Image_2.png]

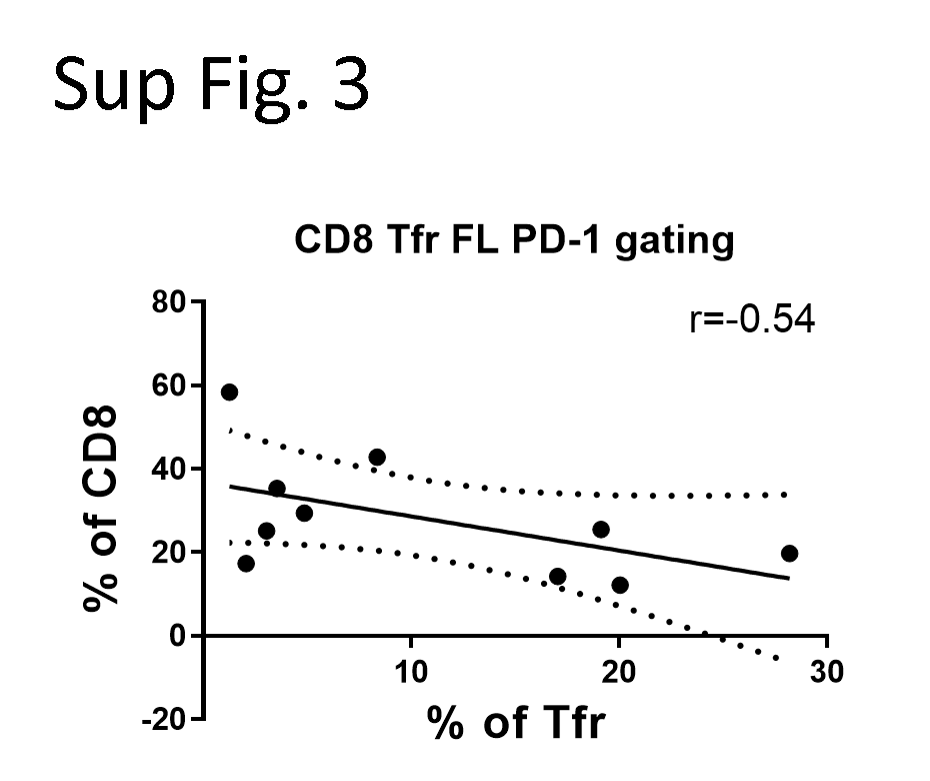

Supplement: Supplementary file 3 [file Image_3.png]
