# Supplementary material for: Follicular lymphoma regulatory T-cell origin and function
Source: Front Immunol. 2024 May 10;15:1391404. doi: 10.3389/fimmu.2024.1391404 (PMC11116630; doi:10.3389/fimmu.2024.1391404)
Supplement: Supplementary file 4 [file DataSheet_1.docx]

**Supplemental material and methods**

**Cell subset isolation**

For CD4^+^ T cell isolation: first, CD19^+^/CD14^+^/CD8^+^/CD16^+^ cell populations were targeted by their dedicated mouse anti-human monoclonal antibody (Beckman Coulter, Villepinte, France; highlighted by *** symbols in supplemental table) followed by an incubation with goat anti-mouse magnetic beads (Miltenyi, Gladsbach, Germany). Undesired cells were bound to the magnetic column of MACS sorter (Miltenyi) and enriched CD4^+^ T cell suspension eluted. Retrieved cells were then stained and sorted according to the following phenotype using a FACSAria flow cytometer:

- Tfh (CD3^+^ CD4^+^ CD45RA^-^ CXCR5^hi^ ICOS^+^ CD25^-^),
- Tfr (CD3^+^ CD4^+^ CD45RA^-^ CXCR5^+^ ICOS^+^ CD25^hi^),
- non-follicular Tregs (CD3^+^ CD4^+^ CXCR5^-^ ICOS^-^ CD25^+^),
- naïve Tregs (CD3^+^ CD4^+^ CD45RA^+^ CD25^hi^ ICOS^-^ CXCR5^-^),
- memory T cells (CD3^+^ CD4^+^ CD45RA^-^ CXCR5^-^ CD25^-^)

Antibodies used for sorting are highlighted by * symbols in supplemental table.

CD8^+^ T-cell isolation was done by depletion of CD19^+^/CD14^+^/CD16^+^ cells using a MACS sorter, followed by CD8^+^ T-cell FACS sorting (CD3^+^ CD4^-^ CD8^+^ lymphoid cells).

**Flow cytometry characterization of CD4 subsets in tonsils and FL lymph nodes**

Abs used for these analyses are highlighted by ** symbols in supplemental table.

**Microarray analysis**

Biotinylated double strand cDNAs were prepared, starting with amplified total RNA. Following fragmentation and end-labelling, cDNAs were hybridized on Human Transcriptome Array 2.0 chip (Affymetrix, Santa Clara, CA). Raw data were controlled with Affymetrix Expression Console software version 1.4.1 and normalized using Robust Multi-array Average (RMA) algorithms with default settings at the gene and exon levels. Correlation matrix were obtained from unsupervised hierarchical clustering of the different gene sets.

**Methylome analysis**

To exclude technical and biological biases that might produce false results in further analyses, we developed and optimized an analysis pipeline with several filters (*i.e.* CpGs with low detection p-values, sex-specific and individual-specific methylation or overlapping with SNPs). Taking into account the different performance of Infinium I and Infinium II assays, we used the subset-quantile within array normalization (SWAN) that corrects technical differences between the Infinium I and II assay designs, and produces a smoother overall beta value distribution. A principal component analysis of the normalized data was performed using R as unsupervised hierarchical clustering.

**Repertoire study**

Library preparation

Briefly, the following primer: TCA-GGC-AGT-ATC-TGG-AGT-CAT-T was used with the SMARTER II A oligonucleotide for reverse transcription. Then, 35 cycles of PCR with the DNA polymerase MyFi (Bioline Meridian) and the following primers: TCGTCGGCAGCGTCAGATGTGTATAAGAGACAGGGGCAAGCAGTGGTATCAACGCAGAGT and GTCTCG-TGGGCTCGGAGATGTGTATAAGAGACAGTGATGGCTCAAACACAGCGACCT was done. PCR products were then purified by the use of magnetic beads (Agilentcount CleanSEQ, Beckman Coulter). Libraries were tagged and quantified by qPCR against the tag.

Repertoire data analysis

Briefly, output files were parsed using in-house R script to determine clonotype abundance and community. All reads sharing the same V and J genes and a highly similar CDR3 sequence were identified as originating from the same clonotype. We then calculated the relative frequency of each clonotype and quantified similarities between repertoires, we used Horn-Morisita index, using the following formula:

$$C_{H}=\frac{2\sum_{i=1}^{S} x_{i}y_{i}}{\left( \frac{\sum_{i=1}^{S} x_{i}^{2}}{X^{2}}+\frac{\sum_{i=1}^{S} y_{i}^{2}}{Y^{2}} \right)XY}$$

**Immunofluorescence**

FFPE tissue blocks were transferred onto plus-charged slides. Mouse anti-human CD25 (Thermo scientific, clone OX-39), rabbit anti-human CD8 (Abcam, clone 4055) and rabbit anti-human FOXP3 (SP97 Bioscience, clone M3972) antibodies were used, followed by incubation with primary antibody and corresponding HRP secondary antibody. Revelations were done on the same section by three sequential rounds of staining each including a heat deactivation step. After sequential reactions, sections were counterstained with DAPI and coverslipped using Fluoromount (Enzo Life Sciences, Farmingdale, NY, USA).

**Supplemental table**

**Supplemental table 1:** Clinical details of follicular lymphoma lymph nodes used in this study

| Company | Description | Clone | Cat. number |
| --- | --- | --- | --- |
| Becton Dickinson® | Anti-human CD45RA BV421* | HI100 | 562885 |
|  | Streptavidin PE-Cy7* |  | 557598 |
|  | Anti human CD25 PE-CF594* | M-A251 | 562403 |
|  | Anti human CD4 BV510* | L200 | 562970 |
|  | Anti human CD3 PE-CF594 ** | UCHT1 | 562280 |
|  | Streptavidin BV711** |  | 563262 |
|  | Anti human Foxp3 A488** | 259D/C7 | 560047 |
|  | Anti-human CD7 BV510 | M-T701 | 563650 |
|  | Anti human Bcl6 PE**  Anti human CD25 BV421**  Anti human CD19 BV510**  Annexin V FITC** | K112-91  M-A251  SJ25C1 | 561522  562442  562953  556420 |
| Beckman Coulter® | Anti-human CD8beta FITC* | 2ST8.5H7 | B42025 |
|  | Anti-human CD8*** | B9.11 | IM0102 |
|  | Anti-human CD14*** | RMO52 | IM0643 |
|  | Anti-human CD16*** | 3G8 | IM0813 |
|  | Anti-human CD19*** | J3-119 | IM1313 |
|  | Anti-human CD3 FITC * | UCHT1 | A07746 |
| Life Technologies® | Anti-Human CD278 (ICOS) Biotin*/** |  | Custom CV001/16/01 |
|  | Transcription Factor Buffer set** |  | 00-5523-00 |
|  | Fixable Yellow Dead Cell Stain Kit** |  | L34959 |
|  | Anti human PD1 PC7** | J105 | 25-2799-42 |
|  | Cell Trace Far Red Kit ** |  | C34564 |
| R&D systems® | Anti-human CXCR5 PE** | 51505 | FAB190P |
|  | Anti-human IL-1 RII PE** | 34141 | FAB663P |
|  | Anti-human CXCR5 APC* | 51505 | FAB190A |
| Miltenyi® | Anti mouse Ig microbeads*** |  | 130-048-401 |
|  | Anti humanCD185 (CXCR5)-APC** | REA103 | 130-098-422 |
| Biolegend® | Anti-human CD4 APC/Fire 750** | SK3 | 344638 |
| Agilent® | Anti-human Ki67** | MIB-1 | F726801-8 |

**Supplemental table 2:**

Table indicating for the different consumables the brand, product name, clone when applicable and the catalog number. Symbols are referring to their usage: cell sorting (*), phenotyping/functional assay (**), cell depletion (***) as described in supplemental materials and methods.

**Supplemental table 3:** Compensation matrix belonging to phenotyping experiments

**Supplemental Figures**

**Supplemental Figure 1**

**A) left panels:** **up:** Illustrating plot depicting CD4^+^CXCR5^+^CD25^+^PD-1^+^ T cells, **down:** CD4^+^CXCR5^+^CD25^+^ICOS^+^ T cells expression of Foxp3 and CD25. **Right Panels: up:** percentage of Foxp3^+^ cells among CD4^+^CXCR5^+^CD25^hi^PD1^+^ T cells. 74% of CD4^+^CXCR5^+^CD25^hi^PD1^+^ T cells (mean 74%±16.52) were Foxp3^+^ and 85% when only red squares were considered.  **down:** percentage of Foxp3^+^ cells among CD4^+^CXCR5^+^CD25^hi^ICOS^+^ T cells, 86% of cells were Foxp3^+^ (mean 86%±13.18) and 94% when only red squares were considered. **B):** Ratio of mean of fluorescence intensity of CD25, CXCR5, ICOS and PD-1 based on: intensity of CD4^+^CXCR5^+^CD25^+^ICOS^+^ cells, Tfh or non-follicular Tregs / intensity of non-follicular-non Treg-T cells (CD4^+^ CXCR5^-^ Foxp3^-^ CD25^-^ ICOS^-^ T cells). **First panel:** CXCR5^+^ CD25^+^ ICOS^+^ T cells (mean= 22.77±18.65) and Tregs (mean= 12.08± 9.115) expressed significantly more CD25 than Tfh (mean= 0.898±0.387) (CXCR5^+^ CD25^+^ ICOS^+^ vs Tfh, p<0.0001, Tregs vs Tfh, p<0.05). **Second panel:** CXCR5^+^ CD25^+^ ICOS^+^ T cells (mean= 15.51±5.797) and Tfh cells (mean= 23.81± 10.35) expressed significantly more CXCR5 than Tregs (mean= 1) (CXCR5^+^ CD25^+^ ICOS^+^ vs Tregs, p<0.0001, Tfh vs Tregs, p<0.05). **Third panel:** Like Tfh (mean=13.87± 12.15), CXCR5^+^ CD25^+^ ICOS^+^ T cells (mean=20.22± 26.57) expressed significantly higher level of ICOS compared to Tregs (mean=2.159± 1.064) (CXCR5^+^ CD25^+^ ICOS^+^ T cells vs Tregs: p<0.005, Tfh vs Tregs: p<0.01). **Fourth panel:** Unlike Tfh (mean=92.77± 30.74), CXCR5^+^ CD25^+^ ICOS^+^ T cells (mean=23.65± 12.89) expressed similar levels of PD-1 than Tregs (mean=1.705± 0.3423).

**Supplemental Figure 2**

**A):** Heatmap of the top 20 differentially expressed genes from transcriptomic analysis of sorted tonsil Tfh (Tons-Tfh), tonsil Tregs (Tons-Treg), FL Tfh and FL Tfr. **B):** Comparison of mouse and human Tfr signatures. Mouse Tfr signature from Linterman *et al*.^21^ was compared to human orthologs, and hypergeometric test was done to quantify the overlap significance. Human and mouse Tfr signatures significantly overlap (p= 1.025 e-21). **C):** Methylation level of several residues belonging to CTLA4 (first panel), Foxp3 (second panel), IKZF4 (third panel) and TNFRSF9 (fourth panel) demonstrating that these genes were preferentially demethylated in tonsil Tregs and FL Tfr compared to tonsil and FL Tfh, tonsil and FL memory T cells.

**Supplemental Figure 3**

Correlation between Tfr and CD8^+^ T cell frequencies with Tfr defined as CD4^+^CXCR5^+^ ICOS^+^CD25^+^ T cells and CD8^+^ T cells defined as CD3^+^ CD4^-^ cells.
